# Deep Brain Stimulation and Thalamotomy for the Treatment of Dystonia Acquired by Moyamoya Disease with Stroke

**DOI:** 10.5334/tohm.73

**Published:** 2020-06-18

**Authors:** Yunhao Wu, Daoqing Su, Yuhan Wang, Hongxia Li, Chencheng Zhang, Bomin Sun, Dianyou Li, Yiwen Wu

**Affiliations:** 1 Department of Functional Neurosurgery, RuiJin Hospital, Shanghai Jiao Tong University School of Medicine, Shanghai, CN; 2 Department of Neurosurgery, Liaocheng People’s Hospital, Liaocheng Clinical School of Shandong First Medical University, Shandong, CN; 3 Department of Neurology, RuiJin Hospital, Shanghai Jiao Tong University School of Medicine, Shanghai, CN

**Keywords:** Moyamoya disease, stroke, deep brain stimulation, thalamotomy

## Abstract

**Background:**

Moyamoya disease (MMD) is a type of chronic cerebrovascular disease. Currently, revascularization surgery including direct/indirect procedure is recommended for symptomatic patients. However, some patients still respond poorly to the treatment or develop secondary symptoms.

**Case report:**

We report the first case of an MMD patient treated with deep brain stimulation (DBS) and thalamotomy. Symptoms of dystonia due to hemorrhage in the thalamus responded poorly to revascularization surgery, but were considerably alleviated by stereotactic neurosurgery.

**Discussion:**

Our case report provides a potential strategy for management of refractory symptomatic MMD patients with dystonia and also supports the combined efficacy of DBS with thalamotomies.

**Highlights:**

Approximately 30% of patients with Moyamoya disease (MMD) presenting movement symptoms do not respond well to revascularization surgery. We reported an MMD patient treated with deep brain stimulation (DBS) and thalamotomy with significant dystonia and dystonic tremor symptom amelioration. It indicates that DBS or stereotactic lesioning might be a potential treatment for the refractory movement symptoms of MMD.

## Introduction

Moyamoya disease (MMD) is a cerebrovascular disease characterized by chronic progressive stenosis of bilateral supraclinoid internal carotid arteries and an abnormal vascular network at the base of the brain. Ischemic symptoms such as transient ischemic attacks are the most common clinical manifestations of MMD, while involuntary movements, usually observed among children, including chorea, dystonia, and dyskinesia are comparatively rare in MMD patients. The latter descriptive features are partially or minimally responsive to medications such as anticholinergic agents and baclofen [1]. Currently, revascularization surgery including direct/indirect procedure is recommended for symptomatic MMD patients. However, some patients still showed unsatisfactory symptom remission [2]. Here, we report a patient with acquired dystonia caused by brain hemorrhage secondary to MMD. She initially underwent revascularization surgery and was subsequently treated with unilateral ventral intermediate nucleus (VIM)/ventral oral nucleus (VOA) radiofrequency thalamotomy and globus pallidus internus (GPi) deep brain stimulation (DBS), which successfully alleviated her dystonic symptoms.

## Case description

**Figure 1:**
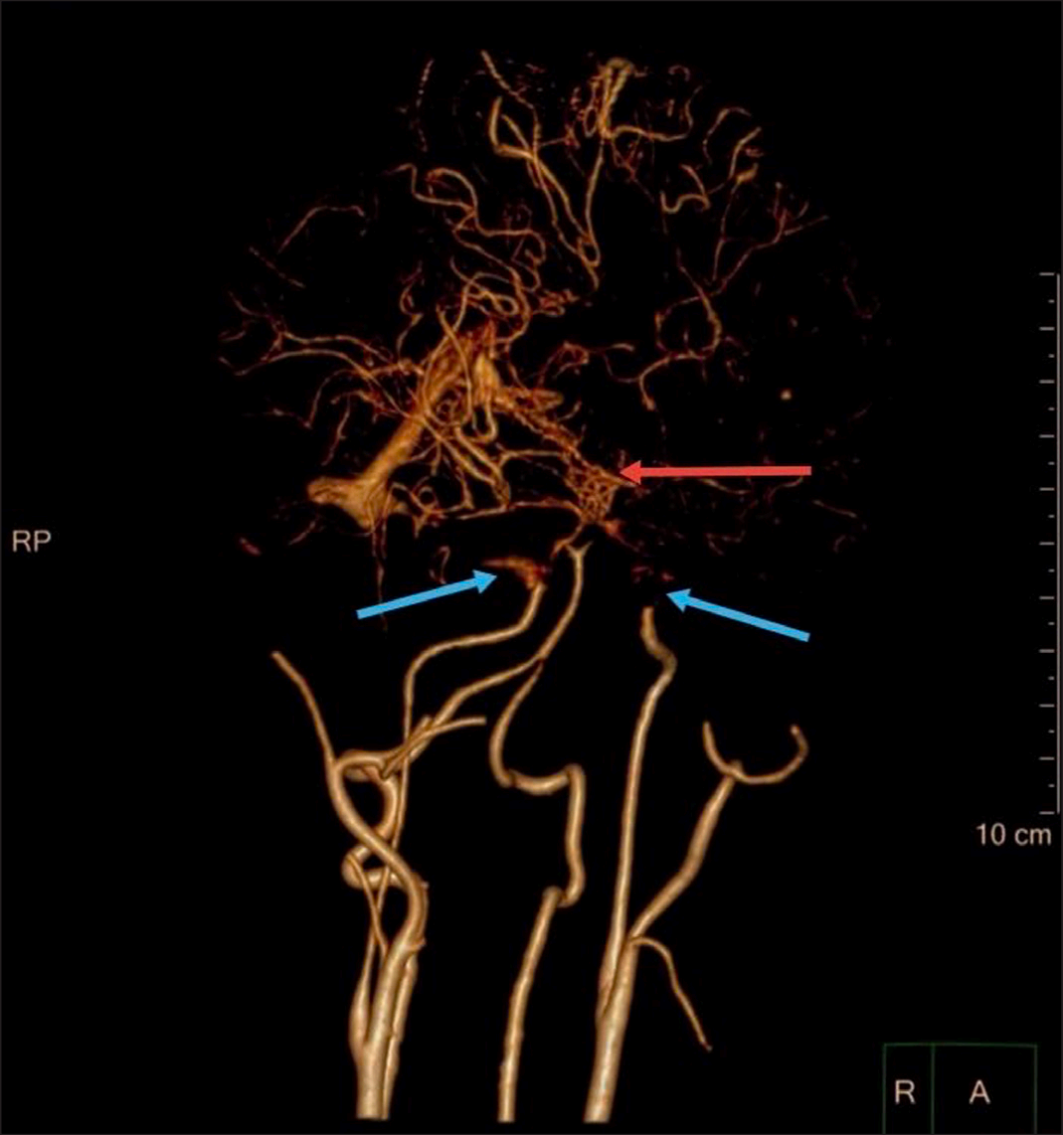
Occlusive changes at bilateral distal internal carotid arteries (ICA), beginning portions of middle cerebral artery (MCA), and anterior cerebral arteries (ACA) (blue arrows). An abnormal vascular network at the base of the brain (“puff of smoke” sign) (red arrow).

**Figure 2:**
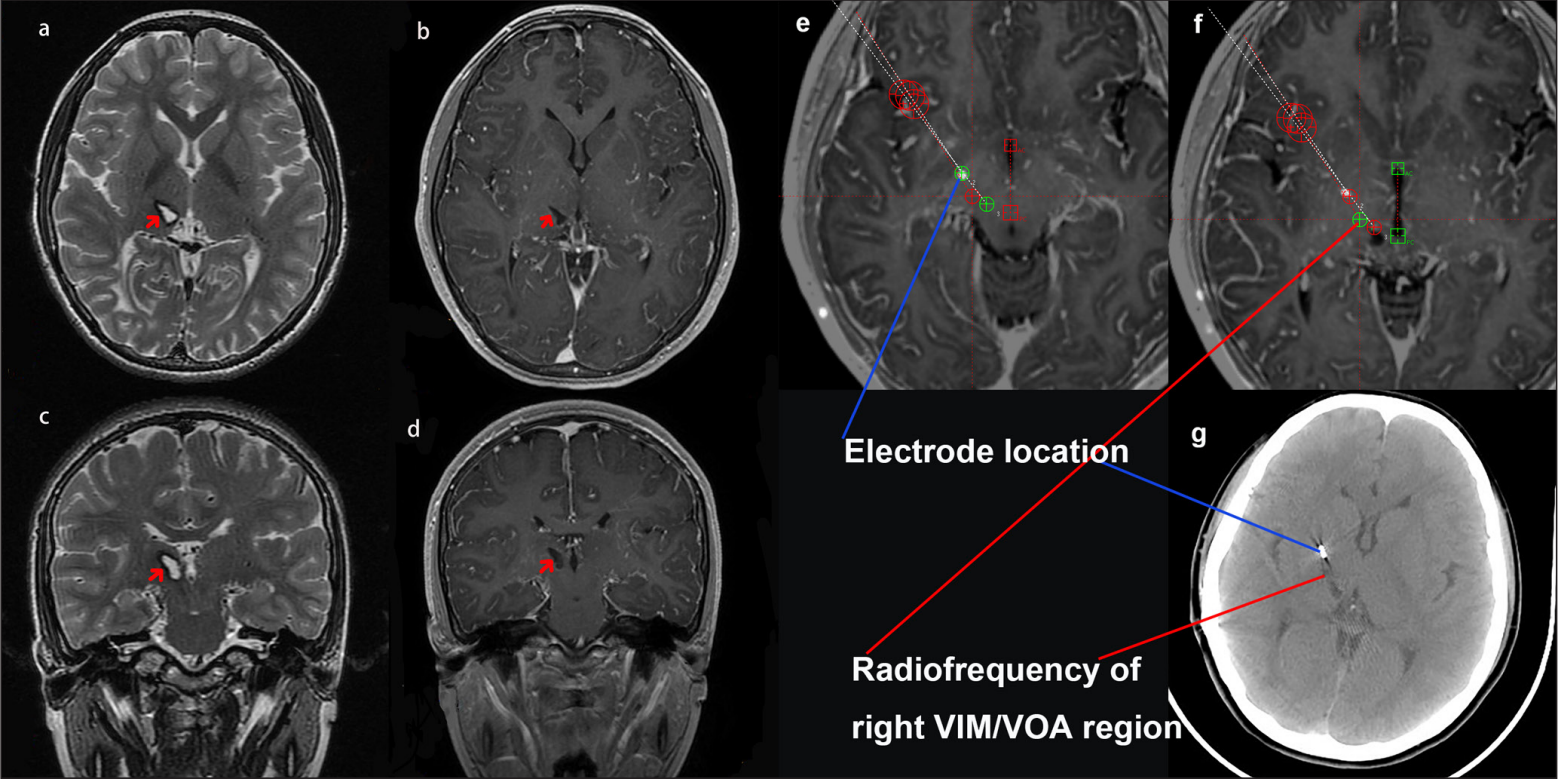
a–d (a and b: axial view; c and d: coronal view): Brain magnetic resonance imaging before stereotactic surgery revealing abnormal signal intensities in the right thalamus (red arrows). e–g: Stereotactic targeting images. Electrode location is marked with a blue line, and ventral intermediate nucleus/ventral oral nucleus radiofrequency lesion is marked with a red line.

The patient was a 15-year-old, right-handed girl who has experienced progressive involuntary movement of her left upper extremity for 5 years. At the age of 10 years, she suddenly developed jerky movements on her left upper limb accompanied by ipsilateral handshaking without triggers. The tremor worsened with activity and seriously affected her fine motor function. She was subsequently sent to a local hospital. Physical examinations showed increased muscular tension and reduced muscular strength (grade 4/5) of the patient’s left upper limbs. Computed tomography (CT) images suggested a recent hemorrhage in the right thalamus, and a typical manifestation of “puff of smoke” sign was revealed on CT angiography (CTA) (Figure 1). With the exclusion of other underlying diseases and considering the above imaging manifestations, the patient was subsequently diagnosed with MMD. After the hemorrhage was stabilized, a local neurosurgeon performed an indirect revascularization surgery to eliminate the symptoms and decrease future rebleed risk. However, the tremor intensified a year later, accompanied by intermittent dystonic wrist supination and involuntary fist clenching (Video 1). Additionally, she experienced left lower limb weakness, resulting in slight gait instability (Video 3). Disease progression gradually impaired her daily functioning even with adequate and continuous medication including clonazepam, and baclofen. Five years later, she was admitted in our hospital for her refractory symptoms. After confirming her diagnosis through magnetic resonance imaging (MRI) (Figure 2) and CTA, the movement disorder specialist described the symptoms above as dystonia movements. More specifically, the patient was diagnosed with acquired dystonia caused by MMD with stroke.

**Video 1:** **Symptoms before and after surgery.** Comparison of patient’s symptoms before surgery, at 1-month follow-up (deep brain stimulation [DBS]-on), and at 36-month follow-up (DBS-on), recorded with patient’s arms suspended.

Our multidisciplinary DBS team discussed and agreed on a treatment strategy of unilateral VIM/VOA radiofrequency thalamotomy and GPi-DBS. Surgical implantation of the right posteroventral GPi electrode (Mode G101A, PINS Medical, Beijing, China) and unilateral thalamotomy were performed during one operation, with informed consent provided by patient’s guardians for both surgery and video recordings. They acknowledged and fully understood the potential perioperative risks, specifically intracranial hemorrhage. Thalamotomy was performed before electrode implantation, leading to an immediate satisfactory outcome (Videos 1 and 2). The dystonia severity improvement scores based on the Burke–Fahn–Marsden Dystonia Rating Scale (BFMDRS) were 12 points (52.2%) and 5 points (55.6%) in motor and disability scores, respectively, at 1.5-year follow-up visit (Table 1), indicating long-term symptom remission. Additionally, the muscle tone and strength of her left upper limb returned to normal after the surgery. Although slight weakness and tremor of her left hand were still observed, her daily functioning was not affected. At the last follow-up visit, stimulation parameters were optimized as follows: frequency, 160 Hz; pulse width, 60 μsec; amplitude, 3.15 V; and electrode configuration, C+3-. Adverse events were not observed.

**Video 2:** **Right hand’s function tests during operation.** Patient’s performances of writing test and rapid succession movement before and after thalamotomy.

**Table 1:** The dystonia severity evaluated by the Burke–Fahn–Marsden Dystonia Rating Scale at baseline and follow-up visit.

	BFMDRS		General response (%BFMDRS change)

	Motor score	Disability score	

Baseline	23	9	/
1-week follow-up (DBS-on)	19	6	–21.88%
1-month follow-up (DBS-on)	14	6	–37.50%
1-month follow-up (DBS-off)	16	/	/
3-month follow-up (DBS-on)	12	5	–46.88%
9-month follow-up (DBS-on)	12	4	–50.00%
36-month follow-up (DBS-on)	11	4	–53.13%
36-month follow-up (DBS-off)	16	/	/

BFMDRS, Burke–Fahn–Marsden Dystonia Rating Scale. Dystonia severity was evaluated using the Burke–Fahn–Marsden Dystonia Rating Scale at baseline and follow-up visit (1 week, 1 month, 3 months, 9 months, and 36 months). At 1-month and 36-month follow-up visit, the symptoms were recorded and assessed at DBS-off status additionally.

## Discussion

Functional stereotactic neurosurgery has revolutionized the treatment for dystonia following the success achieved in Parkinson’s disease (PD). In summary, our patient had dystonia over the left side of her body, which mainly affected her upper limb, with a presurgical BFMDRS score of 32 points. Her symptoms included rigidity, tremor, and involuntary movement of her left upper limb, causing significant disability. According to her diagnosis, GPi-DBS was a primary consideration because it is safe and effective for medically refractory dystonia, specifically the idiopathic group [3]. Some cases also reported GPi-DBS on posthemorrhagic dystonia with a mean improvement of 42% [4]. However, we considered that GPi-DBS alone would not achieve satisfactory efficacy because of the following reasons: (a) cases of GPi-DBS performed for the treatment of acquired dystonia were limited, varied, and generally worse than those for idiopathic dystonia [5,6], and (b) it usually takes several months or even longer to obtain substantial surgical effects from GPi-DBS, which may not live up to our expectation [7]. These indicated that our patient might not achieve an instant and full symptom alleviation from GPi-DBS. Stereotactic radiofrequency thalamotomy was also proven to be effective for movement disorders [8]. VIM and VOA, nuclei that receive cerebellar and pallidal inputs, respectively, are effective targets in ameliorating tremulous limb and muscle tension [9]. They are often selected as the targets of stereotactic surgery not only for essential tremor and PD but also for dystonia, probably under the rationale of inhibiting motor-related output from the GPi to the thalamus [10].

Although DBS has been increasingly used for dystonia patients, ablative surgery still has not been fully replaced by DBS, considering that they both have advantages and limitations. DBS is a reversible and safe procedure that requires the replacement of pulse generators and batteries. However, this procedure is relatively expensive. Thus, considering that DBS is cost-prohibitive [11], the patient’s parents refused the implantation of electrodes at both GPi and VIM/VOA targets. On the contrary, thalamotomy produces irreversible lesions and may also carry the risks of possible severe complications [12,13,14]. Fortunately, this patient developed unilateral symptoms that did not require bilateral procedures since unilateral thalamotomy largely reduced the adverse event risks [15]. Other limitations of thalamotomy include the risk of symptom recurrence, which means the improvement obtained after thalamotomies may diminish over time [3,15]. In this case, the hemorrhagic lesion of the thalamus presented on the presurgical images was located significantly close to and possibly overlapping the VIM/VOA target, indicating that VIM/VOA thalamotomy alone may not produce permanent and satisfactory efficacy.

Unilateral GPi-DBS combined with VIM/VOA thalamotomy results in less financial burden and less risks of side effects. Additionally, previous cases have reported the remarkable efficacy of both DBS and thalamotomy for severe idiopathic dystonia [16] and combined GPi and VIM-DBS for dystonic tremor [17]. In our case, for patient with acquired hemidystonia presenting mainly with dystonic tremor, we expected combining unilateral GPi-DBS with VIM/VOA thalamotomy to achieve maximized efficacy.

The patient experienced a rapid amelioration after VIM/VOA ablation, which can be observed through the recorded video, and greater than 50% BFMDRS improvement was observed at the last follow-up visit. To confirm the separate and combined efficacy of VIM/VOA thalamotomy lesioning and GPi-DBS, we observed and evaluated the patient under DBS-off status (turn off the stimulator for 24 hours). At 1-month follow-up, the DBS-off BFMDRS motor score (16) was worse than the DBS-on status (14) (present on Video 3) and better than presurgical status (23), supporting the benefits of both thalamotomy and DBS, and the efficacy lasted at least 36 months after the surgery.

**Video 3:** **Gait’s change with DBS off.** Patient’s gait was recorded at deep brain stimulation (DBS)-on state (1-month follow-up) and when the stimulator was turned off 24 hours later. With DBS off, the patient experienced slight left leg weakness and gait instability.

According to previously published reports [18], the efficacy of stereotactic neurosurgeries such as DBS for acquired dystonias varies widely, probably due to various brain lesions involved. In our case, thalamus hemorrhage presented on MRI indicated that dyskinetic symptoms might be generated by basal ganglia-thalamic-cortical circuit dysfunction leading to the disinhibition of the motor cortex. We hypothesized that the underlying DBS and ablation mechanisms are possibly associated with the modulation of such abnormal circuit, which requires further pathophysiological evidences.

To the best of our knowledge, this case reports the first application of stereotactic neurosurgery to movement disorders in MMD and is the first paper to report surgery of GPi-DBS with VIM/VOA thalamotomy for posthemorrhagic dystonia. Approximately 30% of the symptomatic MMD patients respond poorly to the established revascularization surgery [1]. The success rate of stereotactic neurosurgery suggests that this treatment procedure is considered a potential novel strategy for the management of patients with movement symptoms such as dystonia. Meanwhile, it highlights the efficacy produced by multi-target stereotactic neurosurgery. More clinical and prospective studies are required to investigate the efficacy of stereotactic neurosurgery for refractory movement symptoms of MMD patients and the potential role of GPi-DBS combined with VIM/VOA ablation for complex dystonia symptoms.
